# Tr1-Like T Cells – An Enigmatic Regulatory T Cell Lineage

**DOI:** 10.3389/fimmu.2016.00355

**Published:** 2016-09-14

**Authors:** Anna Malgorzata White, David C. Wraith

**Affiliations:** ^1^School of Cellular and Molecular Medicine, University of Bristol, Bristol, UK; ^2^Institute of Immunology and Immunotherapy, University of Birmingham, Birmingham, UK

**Keywords:** Tr-1 T cells, IL-10, CD4^+^ T cell, inhibitory receptors, peripheral tolerance

## Abstract

The immune system evolved to respond to foreign invaders and prevent autoimmunity to self-antigens. Several types of regulatory T cells facilitate the latter process. These include a subset of Foxp3^−^ CD4^+^ T cells able to secrete IL-10 in an antigen-specific manner, type 1 regulatory (Tr1) T cells. Although their suppressive function has been confirmed both *in vitro* and *in vivo*, their phenotype remains poorly defined. It has been suggested that the surface markers LAG-3 and CD49b are biomarkers for murine and human Tr1 cells. Here, we discuss these findings in the context of our data regarding the expression pattern of inhibitory receptors (IRs) CD49b, TIM-3, PD-1, TIGIT, LAG-3, and ICOS on Tr1-like human T cells generated *in vitro* from CD4^+^ memory T cells stimulated with αCD3 and αCD28 antibodies. We found that there were no differences in IR expression between IL-10^+^ and IL-10^−^ T cells. However, CD4^+^IL-10^+^ T cells isolated *ex vivo*, following a short stimulation and cytokine secretion assay, contained significantly higher proportions of TIM-3^+^ and PD-1^+^ cells. They also expressed significantly higher TIGIT mRNA and showed a trend toward increased TIM-3 mRNA levels. These data led us to conclude that large pools of IRs may be stored intracellularly; hence, they may not represent ideal candidates as cell surface biomarkers for Tr1-like T cells.

## CD4^+^IL-10^+^ T Cells – A Heterogeneous Population of Cells with a Suppressive Function

In 1997, Groux et al. described a unique population of CD4^+^ T lymphocytes generated after *in vitro* stimulation of CD4^+^ T cells from the T cell receptor (TCR) transgenic DO11-10 mouse with ovalbumin peptide (OVA) and IL-10 or with IL-10 alone. These OVA-specific CD4^+^ T cells produced high levels of IL-10 and IL-5, moderate levels of IFNγ and TGFβ, low levels of IL-2 and IL-4, and proliferated poorly in response to peptide stimulation (1). High levels of IL-10 suggested a regulatory potential for these cells, since IL-10 is crucial for limiting proinflammatory and autoimmune responses [reviewed in Ref. (2, 3)]. IL-10-deficient mice develop severe colitis, accompanied by tissue damage and excessive inflammation (4). Analysis of this model as well as further studies demonstrated that IL-10 is able to block immune responses at different levels by acting directly and indirectly on both innate and adaptive arms of the immune system [reviewed in Ref. (5)]. As a result, IL-10 can inhibit production of proinflammatory cytokines, antigen presentation, and cell proliferation.

In the original paper, the reconstitution of *SCID* mice with naive CD4^+^ T cells and OVA-specific CD4^+^IL-10^+^ T cell clones resulted in prevention of colitis (1). Later on, several other groups confirmed the antigen-specific suppressive potential of CD4^+^IL-10^+^ T cells *in vivo* using colitis (4), experimental autoimmune encephalomyelitis (EAE) (6–10), collagen-induced arthritis (11), and allergy (12) disease models. Our laboratory has developed an animal model of multiple sclerosis (MS) using a TCR transgenic system. More than 90% of CD4^+^ T cells in transgenic Tg4 mice express a Vβ8.2 TCR specific for the myelin basic protein (MBP) peptide Ac1–9 presented in the context of MHC I-A^u^ (13). This allowed us to conduct detailed phenotypic analysis of antigen-specific CD4^+^IL-10^+^ T cells and identify panel of inhibitory receptors (IRs), which could serve as markers for these cells (7), (see The Need to Discover a Surrogate Marker for Regulatory Tr1-Like T Cells).

CD4^+^IL-10^+^ T cells can also be generated using human peripheral blood mononuclear cells (PBMC) after stimulation with allogeneic monocytes in the presence of IL-10 (1). They produce high levels of IL-10 and low levels of IL-2 and IL-4, after re-stimulation with αCD3 and αCD28 antibodies, and similar to the murine CD4^+^IL-10^+^ T cells can suppress responder CD4^+^ T cells *in vitro* (14–17). These results were obtained using several different protocols for the generation of the CD4^+^IL-10^+^ T cell population. They involved stimulation with a specific subset of antigen-presenting cells, including plasmacytoid dendritic cells (DCs), immature DCs, or tolerogenic DC (1, 18–20); the presence of cytokines, including IL-10, IL-6, IL-21, or IL-27 (21–24); or using antibodies against various costimulatory molecules, such as CD46, CD2, and CD55 (25, 26), as well as vitamin D3 and immunosuppressive drugs (27). Different laboratories adopted a different set of stimuli, which in their experimental setup worked most efficiently to generate high numbers of CD4^+^IL-10^+^ T cells. However, one could question the physiological relevance of such manipulations, because they cannot re-create a complex *in vivo* environment. Also, different protocols result in the emergence of various subpopulations of CD4^+^IL-10^+^ T cells, making characterization of these cells and discovery of specific marker/s of the CD4^+^IL-10^+^ T cell population even more challenging. Despite the heterogeneity of the described human CD4^+^IL-10^+^ populations, these are generally referred to as T regulatory type 1 cells (Tr1). Nevertheless, it is worth noting that to date, it has not been proven that they represent a unique cell lineage; therefore, we will refer them here as *Tr1-like T cells*.

## The Need to Discover a Surrogate Marker for Regulatory Tr1-Like T Cells

The existence of antigen-specific suppressor Tr1-like T cells makes them an appealing target for designing antigen-specific therapies to treat a wide array of autoimmune diseases and to avoid unnecessary and often burdensome side effects associated with conventional immunosuppressive therapies (28). A constitutively expressed surface marker for Tr1-like T cells would allow us to monitor the emergence, numbers, and functionality of these cells. Many groups have tried to identify such a marker in mouse and man (16, 29–32). In 2013, Gagliani et al. postulated that lymphocyte-activation protein 3 (LAG-3) and CD49b are markers for human Tr1-like T cells (17). LAG-3 belongs to a large family of IRs that are upregulated on activated T cells (33). Here, we discuss the significance of the published findings in the context of other relevant IRs.

The *in vivo* data from our laboratory (7) demonstrated that administration of soluble MBP Ac1–9 peptide, using a dose escalation protocol, resulted in abrogation of EAE, which coincided with appearance of antigen-specific CD4^+^IL-10^+^ T cells. A majority of these cells expressed T cell immunoglobulin and mucin domain-3 (TIM-3), T-cell immunoreceptor with Ig and ITIM domains (TIGIT), and 50% of the cells were CD49b^+^, which is in sharp contrast with the expression pattern observed on the IL-10^−^ T cell subset, where all three markers were present in 6–9% of the cells. Programed cell death protein 1 (PD-1) and LAG-3 were found in the majority of CD4^+^ T cells, regardless of IL-10 production. These IRs are involved in several mechanisms regulating T cell signaling [reviewed in Ref. (33, 34)]. PD-1 and TIM-3 bind intracellular mediators as SHIP-1/2 (PD-1), Fyn, and PI3K kinase (TIM-3) to deactivate the downstream signaling molecules, and PD-1 can also induce inhibitory genes that inhibit T cell function. TIGIT and LAG-3 prevent optimal signal transduction at the cell membrane by sequestering counter receptors/ligands together with preventing proper formation of the immunological synapse [reviewed in Ref. (33, 34)]. Importantly, these events are dysregulated not only during autoimmune responses (35–37) but also in tumor formation. PD-1, TIM-3, and LAG-3 are found in T cells isolated from melanoma patients (38–40); therefore, their expression on T cells is also relevant for the development of new anticancer therapies.

PD-1, belonging to the CD28/CTLA-4 family, provides a negative signal following antigen stimulation [reviewed in Ref. (41)]. Depending on the genetic background, PD-1^−/−^ mice develop a range of autoimmune disorders: lupus-like glomerulonephritis in the C57BL/6 strain, autoimmune dilated cardiomyopathy and gastritis in BALB/C, acute type 1 diabetes mellitus (T1DM) in NOD (42), and myocarditis in MRL, suggesting that other genetic, inherent factors act synergistically with PD-1 in each mouse strain (36). Clearly, PD-1 plays an important role in the maintenance of peripheral tolerance, but due to abundant expression on activated T cells, it is unlikely to be a biomarker for Tr1-like T cells.

Apart from PD-1, deficiency in any other of the above-mentioned IRs does not result in the spontaneous development of autoimmune disorders. NOD LAG-3-deficient mice show mild enhancement of T lymphocyte responses, unless crossed with PD-1^−/−^ knockout mice, which causes a lethal myocarditis (36). TIGIT^−/−^ mice are more susceptible to EAE and show only augmented T cell responses when challenged with MOG peptide *in vivo* (37). CD49b deficiency leads to failure of the establishment of memory T cells in the bone marrow (43), but it does not have any profound effect on peripheral tolerance, while TIM-3^−/−^ mice and mice treated with a TIM-3 Ig fusion protein exhibit moderate defects in induction of antigen-specific tolerance [reviewed in Ref. (44)].

Regarding surface expression, most of the above-mentioned IRs are found in cells with regulatory properties. TIGIT is expressed on human Tr1-like T cells (our observation) but also on human T_reg_ Foxp3^+^ cells (45). Furthermore, its presence at the cell surface coincides with increased expression of ICOS, TIM-3, and PD-1 on murine T_reg_ cells (37). TIM-3 is upregulated upon activation *in vitro* in the human T_reg_ subset (46). Recently, it has also been reported that murine CD4^+^CD49b^+^ T cells produce high levels of IL-10 and are potent suppressors of arthritis severity when injected *in vivo* (47).

**Figure 1:**
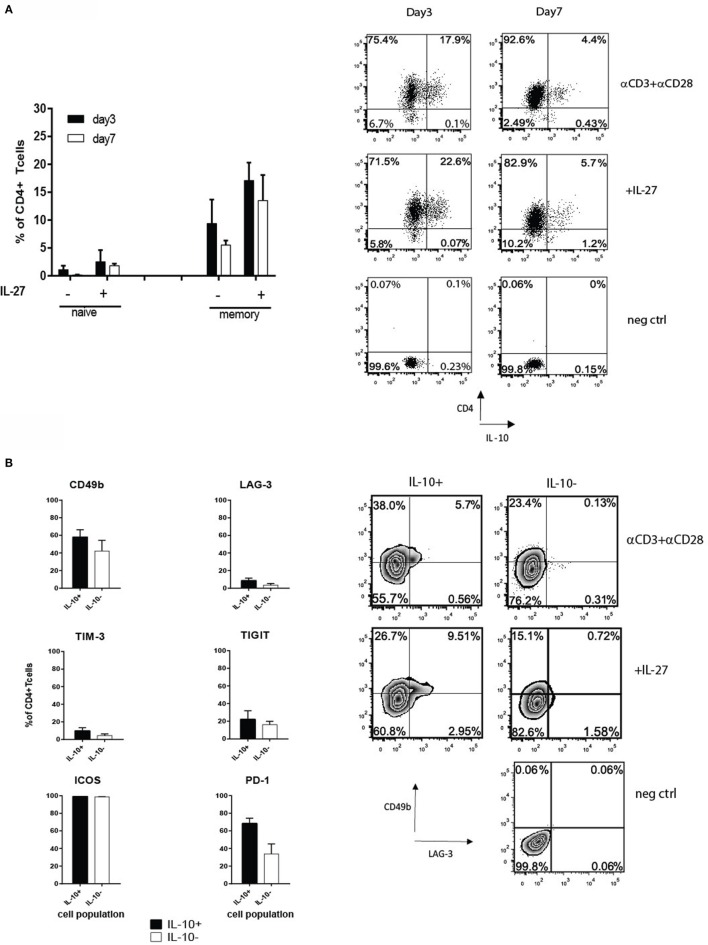
**(A)** Naive and memory CD4^+^ T cells were isolated from PBMC from healthy donors by magnetic selection and stimulated with plate-bound 1 μg/ml αCD3 and 2 μg/ml αCD28 ± 100 ng/ml of IL-27. Intracellular staining for IL-10 was performed on days 3 and 7 after an additional 4-h stimulation with PMA/ionomycin in the presence of Golgi stop. Graphs show the percentages (mean value ± SEM, *n* = 3 donors) of viable CD4^+^IL-10^+^ T cells derived from the naive or memory cell subsets (left panel). A representative dot plot of CD4 and IL-10 staining on memory-derived CD4^+^ T cells on day 7 is shown in the right panel. **(B)** Expression of inhibitory receptors (IRs) on CD4^+^IL-10^+/−^ T cells derived from memory pool after 7 days of cell culture in the presence of 1 μg/ml αCD3 and 2 μg/ml αCD28 examined by flow cytometry. The black bars represent the average percentage of IL-10^+^ and white bars the IL-10^−^ cell fractions, respectively (mean + SEM, *n* = 3 donors). Right panel shows a representative dot plot of CD49b and LAG-3 expression on day 7 by memory CD4^+^IL-10^+/−^ stimulated with αCD3 and αCD28 ± IL-27.

To gain a more detailed understanding of how the expression of PD-1, LAG-3, TIM-3, TIGIT, and ICOS correlates with IL-10 production by human CD4^+^ T cells, we developed a protocol to generate IL-10^+^ CD4^+^ T cells after a different length of stimulation *in vitro*. The first approach involved isolation of memory and naive T cells and stimulating them with αCD3 and αCD28 antibodies in presence or absence of IL-27, known stimuli for Tr1-like T cell generation (21, 22). IL-27 boosted the percentages of CD4^+^IL-10^+^ T cells from 10 to 18% on day 3, which on day 7 decreased to 14% in the presence of IL-27 and 5% in its absence (Figure 1A). However, adding IL-27 did not alter the surface phenotype of Tr1-like T cells (data not shown). Initially, the induction of IL-10 production was accompanied by a slight increase in proportions of cells expressing CD49b and TIM-3 within the IL-10^+^ T cell subset, but this was not statistically significant. By day 7, CD49b was found in similar percentages on both IL-10^+^ and IL-10^−^ T cells (Figure 1B). CD49b and TIM-3 were co-expressed by the IL-10^+^ T cell fraction, but on day 7, CD49b^+^ cells were mainly TIM-3^−^. LAG-3 was expressed by 8.6% of IL-10^+^ T cells on day 7 (Figure 1B), and among these cells, 30% co-expressed TIM-3. The co-expression of LAG-3 and CD49b was observed only on a small proportion of IL-10^+^ subset (Figure 1B, right panel). The proportion of cells expressing TIGIT was similar between IL-10^+^ and IL-10^−^ subsets of CD4^+^ T cells (Figure 1B), and approximately half of them co-expressed TIM-3. Our observations led us to conclude that none of the tested IRs are exclusively expressed on IL-10-producing T cells, and their expression is dynamic, changing over the time course of cell culture.

**Figure 2:**
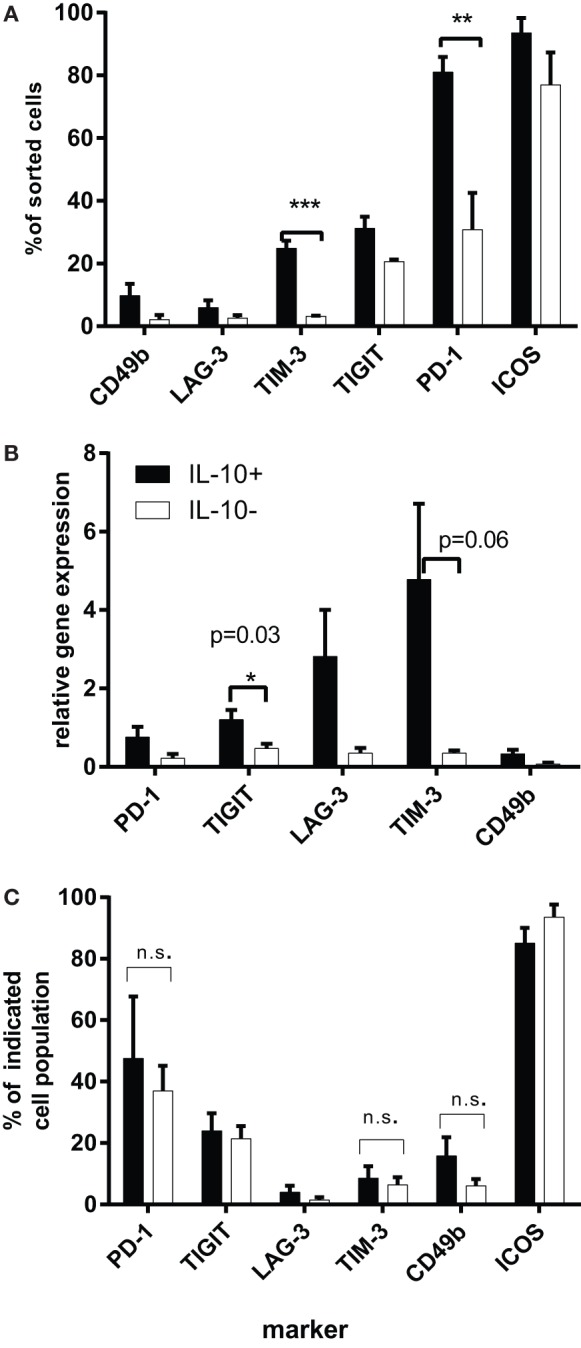
**(A)** The expression of IRs on purified CD4^+^IL-10^+^/IL-10^−^ T cells. Magnetically sorted CD4^+^ T cells were cultured for 16 h in the presence of 1 μg/ml αCD3 and 2 μg/ml of αCD28 antibodies, then harvested, subjected to IL-10 cytokine secretion assay, and sorted by flow cytometry according to their IL-10 expression. Graphs represent the percentages of IL-10^+^ or IL-10^−^ T cells, expressing each IR determined by flow cytometry (*n* = 4). Purified CD4^+^IL-10^+/−^ T cells were rested for 48 h in the presence of 60 U/ml IL-2 and then were restimulated for 4 h with αCD3 and αCD28 antibodies. **(B)** mRNA levels of IRs on sorted CD4^+^IL-10^+/−^ T cells. Purified CD4^+^IL-10^+/−^ T cells were rested for 48 h in the presence of 60 U/ml IL-2 and then were restimulated for 4 h with αCD3 and αCD28 antibodies. Graphs show mean gene expression levels as relative values compared to HPRT-1 (*n* = 4). **(C)** Expression of IRs on CD4^+^IL-10^+/−^ T cell fraction at the point of RNA isolation as evaluated by flow cytometry. Figure shows percentages of viable CD4^+^IL-10^+/−^ T cells expressing the indicated marker (mean + SEM, *n* = 4). The significance has been analyzed using *t* test.

These results were very different from the data generated *in vivo* in the tolerance model (7). Therefore, we used a modified version of the protocol previously developed in our laboratory (48), which involved the *ex vivo* isolation of IL-10^+^ CD4^+^ T cells, a short stimulation of unfractionated CD4^+^ T cells with αCD3 and αCD28 antibodies, followed by IL-10 cytokine secretion assay to allow sorting of IL-10^+^ cells. This strategy minimized the manipulation of cells *in vitro* but still allowed us to obtain a sufficient number of CD4^+^IL-10^+^ T cells for analysis (3–5% of total CD4^+^ T cells). The phenotype of highly purified IL-10^+^ cells differed significantly from the IL-10^−^ subpopulation. TIM-3 expression was significantly higher on IL-10^+^ T cells as compared to the IL-10^−^ subset (*p* = 0.0008) and was present in approximately 25% of CD4^+^ T cells, while 80% of CD4^+^IL-10^+^ T cells expressed PD-1 (*p* = 0.007), which was significantly higher when compared to the IL-10^−^ fraction. Within the PD-1^+^ T cell population, the percentages of CD49b^+^ and LAG-3^+^ cells were lower, both below 10% and although higher than the IL-10^+^ subset, the differences were not statistically significant (Figure 2A).

To correlate the surface phenotype with RNA levels, we performed RT-PCR on restimulated IL-10^+^ and IL-10^−^ cells, which were previously rested for 48 h in the presence of human recombinant IL-2. RT-PCR analysis demonstrated a significant increase in TIGIT expression among IL-10^+^ T cells (*p* = 0.03) and a trend toward higher levels of TIM-3 mRNA levels among IL-10^+^ T cells as compared to the IL-10^−^ T cell subset (Figure 2B), pointing to these two markers as preferential for Tr1-like T cells. However, our flow cytometry analysis of surface IR levels performed at the same time point resulted in a different pattern of expression. There were no statistically significant differences in TIM-3 expression between IL-10^+^ and IL-10^−^ T cells (approximately 10%), similar to LAG-3^+^ (5%), much lower as compared to relative RNA levels. By contrast, TIGIT^+^IL-10^+^ T cells comprised 25% and PD-1^+^ 50% of IL-10^+^ T cells, while their RNA levels were lower than those of TIM-3 and LAG-3. It is also important to note that there were no noticeable differences in the percentages of TIM-3, TIGIT, and PD-1 between IL-10^+^ and IL-10^−^ T cell subsets at this time point (Figure 2C). This result could be explained by the fact that these IRs are stored intracellularly (49–51) and released to the surface with different kinetics; so although their mRNA is upregulated, this may contribute to intracellular pools rather than cell surface expression of the markers. It has been previously shown that large pools of LAG-3 are stored intracellularly (49), and we were able to detect large proportions of intracellular LAG-3 in both IL-10^+^ and IL-10^−^ T cells (data not shown). It is possible that the kinetics of LAG-3 release to the cell surface correlates with the suppressive phenotype of the cells. In the same way, surface expression of TIM-3, known to reside in the Golgi apparatus and endoplasmic reticulum (50), could be differentially regulated on the IL-10^+^ as compared to the CD4^+^IL-10^−^ subpopulation. There is also evidence for altered regulation of TIM-3 expression in acute myeloid leukemia, where the majority of TIM-3 is expressed on the surface of PMBC as compared to healthy individuals, where TIM-3 is mainly detected intracellularly (51).

## Beyond IRs

Due to the lack of any firm evidence demonstrating an exclusive IR marker for Tr1-like T cells, emerging evidence points toward new molecules that might serve as their biomarkers. In 2015, Blumberg’s group published a very elegant study in which they demonstrated that surface expression of TIM-3 is regulated by carcinoembryonic antigen cell adhesion molecule 1 (CEACAM-1), which has the ability to form a heterodimer with TIM-3 in human and murine CD4^+^ T cells (50). Furthermore, lower proportions of tumor-infiltrating murine CD4^+^ and CD8^+^ T cells produced IL-10 after co-blockade of CEACAM-1 and TIM-3 *in vivo* (50). Equally, one could speculate that CEACAM-1 could also regulate surface expression of TIM-3 on Tr1-like T cells; however, the CEACAM-1 expression on Tr1-like T cells has not yet been studied. It is known that this molecule is expressed on a small population of resting CD4^+^ T cells in humans and mice (52); hence, it could be a potential candidate for a biomarker for Tr1-like T cells.

The second putative candidate is Granzyme B. Previous studies have shown that human and murine CD4^+^ T cells, which acquire a Tr-1-like phenotype, express Granzyme B (53–55). A recent publication by Schmetterer et al. demonstrates that human CD4^+^ T cells transduced with the active form of STAT3 produce higher levels of IL-10 and Granzyme B, which was responsible for the suppressive activity of these cells (56). It is worthwhile to point out that the cells did not display elevated levels of CD49b and LAG-3 (56). Interestingly, blocking CEACAM1 increased the cytolytic function of human CD8^+^ T cells (52); hence, this molecule if expressed on CD4^+^IL-10^+^ T cells could influence their cytotoxic function by regulating Granzyme B expression.

The third possible candidate to serve as a marker for Tr1-like T cells is class I-restricted T cell-associated molecule (CRTAM) expressed on both CD4^+^ and CD8^+^ T cells upon activation. This molecule was upregulated on Tr1-like T cells as a result of tolerance induction after administration of escalating doses of MBP peptide (7). Recently, Saito’s group demonstrated that CRTAM is expressed on a specific subset of CD4^+^ T cells, which are characterized by high production of IFNγ, expression of Granzyme B, and Eomes after TCR activation, and can develop cytotoxic properties in both mice and humans (57). A comparison of the phenotype of CD4^+^CRTAM^+^ T cells with Tr1-like T cells in relation to expression of Granzyme B and CEACAM-1 would provide an insight into the functional differences within a heterogeneous subset of human Tr1-like T cells, especially given that, according to our observations, 50% of these cells expressed IFNγ.

In summary, our phenotypic analyses suggest that none of the analyzed IRs can be described as surrogate markers for Tr1-like T cells. Ideally, such a biomarker would be a stable, constitutively expressed cell surface molecule, easily detected on freshly isolated human CD4^+^ T cells. In the quest to identify it, more detailed analyses using RNA profiling and unbiased proteomics together with studies of epigenetic changes at the IL-10 promoter should be performed. Our laboratory analyzed changes in histone H3 modification at the IL-10 promoter and found similar epigenetic changes in mouse and human CD4^+^IL-10^+^ T cells (58). However, Dong et al. found limited epigenetic changes in the status of human IL-10 promoter and a lack of functional memory for IL-10 re-expression in cultured IL-10 secreting cells (59). It is clear that all T cell subsets can secrete IL-10 under certain circumstances (3). Therefore, the question as to whether Tr1 cells constitute a distinct lineage remains open and requires further investigation.

## Ethics Statement

NRES Committee North West – Greater Manchester West, Ethical Permission 14/NW/0152. The study did not require consent because it was anonymized study, and we used lymphocyte cones purchased from the NHS blood bank in Bristol, United Kingdom.

## Author Contributions

AW performed experimental work and wrote the manuscript. DW coordinated the experimental work and co-wrote the manuscript.

## Conflict of Interest Statement

The authors declare that the research was conducted in the absence of any commercial or financial relationships that could be construed as a potential conflict of interest.

## References

[1] Groux H O’Garra A Bigler M Rouleau M Antonenko S De Vries JE A CD4+ T-cell subset inhibits antigen-specific T-cell responses and prevents colitis. Nature (1997) 389:737–42. 10.1038/39614 9338786

[2] Moore KW De Waal Malefyt R Coffman RL O’Garra A. Interleukin-10 and the interleukin-10 receptor. Annu Rev Immunol (2001) 19:683–765. 10.1146/annurev.immunol.19.1.683 11244051

[3] Ng TH Britton GJ Hill EV Verhagen J Burton BR Wraith DC. Regulation of adaptive immunity; the role of interleukin-10. Front Immunol (2013) 4:129. 10.3389/fimmu.2013.00129 23755052 PMC3668291

[4] Kuhn R Lohler J Rennick D Rajewsky K Muller W. Interleukin-10-deficient mice develop chronic enterocolitis. Cell (1993) 75:263–74. 10.1016/0092-8674(93)80068-P 8402911

[5] Saxena A Khosraviani S Noel S Mohan D Donner T Hamad AR. Interleukin-10 paradox: a potent immunoregulatory cytokine that has been difficult to harness for immunotherapy. Cytokine (2015) 74:27–34. 10.1016/j.cyto.2014.10.031 25481648 PMC4454631

[6] Gabrysova L Wraith DC. Antigenic strength controls the generation of antigen-specific IL-10-secreting T regulatory cells. Eur J Immunol (2010) 40:1386–95. 10.1002/eji.200940151 20162554 PMC3466465

[7] Burton BR Britton GJ Fang H Verhagen J Smithers B Sabatos-Peyton CA Sequential transcriptional changes dictate safe and effective antigen-specific immunotherapy. Nat Commun (2014) 5:4741. 10.1038/ncomms5741 25182274 PMC4167604

[8] Nicolson KS O’Neill EJ Sundstedt A Streeter HB Minaee S Wraith DC. Antigen-induced IL-10+ regulatory T cells are independent of CD25+ regulatory cells for their growth, differentiation, and function. J Immunol (2006) 176:5329–37. 10.4049/jimmunol.176.9.5329 16622000 PMC2629539

[9] Verhagen J Gabrysova L Shepard ER Wraith DC. Ctla-4 modulates the differentiation of inducible Foxp3+ Treg cells but IL-10 mediates their function in experimental autoimmune encephalomyelitis. PLoS One (2014) 9:e108023. 10.1371/journal.pone.0108023 25238105 PMC4169598

[10] Bettelli E Das MP Howard ED Weiner HL Sobel RA Kuchroo VK. IL-10 is critical in the regulation of autoimmune encephalomyelitis as demonstrated by studies of IL-10- and IL-4-deficient and transgenic mice. J Immunol (1998) 161:3299–306. 9759845

[11] Johansson AC Hansson AS Nandakumar KS Backlund J Holmdahl R. IL-10-deficient B10.Q mice develop more severe collagen-induced arthritis, but are protected from arthritis induced with anti-type II collagen antibodies. J Immunol (2001) 167:3505–12. 10.4049/jimmunol.167.6.3505 11544344

[12] Akdis CA Blaser K. IL-10-induced anergy in peripheral T cell and reactivation by microenvironmental cytokines: two key steps in specific immunotherapy. FASEB J (1999) 13:603–9. 10094921 10.1096/fasebj.13.6.603

[13] Liu GY Fairchild PJ Smith RM Prowle JR Kioussis D Wraith DC. Low avidity recognition of self-antigen by T cells permits escape from central tolerance. Immunity (1995) 3:407–15. 10.1016/1074-7613(95)90170-1 7584132

[14] Levings MK Roncarolo MG. T-regulatory 1 cells: a novel subset of CD4 T cells with immunoregulatory properties. J Allergy Clin Immunol (2000) 106:S109–12. 10.1067/mai.2000.106635 10887343

[15] Vieira PL Christensen JR Minaee S O’Neill EJ Barrat FJ Boonstra A IL-10-secreting regulatory T cells do not express Foxp3 but have comparable regulatory function to naturally occurring CD4+CD25+ regulatory T cells. J Immunol (2004) 172:5986–93. 10.4049/jimmunol.172.10.5986 15128781

[16] Haringer B Lozza L Steckel B Geginat J. Identification and characterization of IL-10/IFN-gamma-producing effector-like T cells with regulatory function in human blood. J Exp Med (2009) 206:1009–17. 10.1084/jem.20082238 19414553 PMC2715038

[17] Gagliani N Magnani CF Huber S Gianolini ME Pala M Licona-Limon P Coexpression of CD49b and LAG-3 identifies human and mouse T regulatory type 1 cells. Nat Med (2013) 19:739–46. 10.1038/nm.3179 23624599

[18] Pletinckx K Vaeth M Schneider T Beyersdorf N Hunig T Berberich-Siebelt F Immature dendritic cells convert anergic nonregulatory T cells into Foxp3- IL-10+ regulatory T cells by engaging CD28 and CTLA-4. Eur J Immunol (2015) 45:480–91. 10.1002/eji.201444991 25382658

[19] Jonuleit H Schmitt E Schuler G Knop J Enk AH. Induction of interleukin 10-producing, nonproliferating CD4(+) T cells with regulatory properties by repetitive stimulation with allogeneic immature human dendritic cells. J Exp Med (2000) 192:1213–22. 10.1084/jem.192.9.1213 11067871 PMC2193357

[20] Amodio G Gregori S. Human tolerogenic DC-10: perspectives for clinical applications. Transplant Res (2012) 1:14. 10.1186/2047-1440-1-14 23369527 PMC3560992

[21] Murugaiyan G Mittal A Lopez-Diego R Maier LM Anderson DE Weiner HL. IL-27 is a key regulator of IL-10 and IL-17 production by human CD4+ T cells. J Immunol (2009) 183:2435–43. 10.4049/jimmunol.0900568 19625647 PMC2904948

[22] Pot C Jin H Awasthi A Liu SM Lai CY Madan R Cutting edge: IL-27 induces the transcription factor c-Maf, cytokine IL-21, and the costimulatory receptor ICOS that coordinately act together to promote differentiation of IL-10-producing Tr1 cells. J Immunol (2009) 183:797–801. 10.4049/jimmunol.0901233 19570826 PMC2768608

[23] Wang H Meng R Li Z Yang B Liu Y Huang F IL-27 induces the differentiation of Tr1-like cells from human naive CD4+ T cells via the phosphorylation of STAT1 and STAT3. Immunol Lett (2011) 136:21–8. 10.1016/j.imlet.2010.11.007 21115047

[24] Jin JO Han X Yu Q. Interleukin-6 induces the generation of IL-10-producing Tr1 cells and suppresses autoimmune tissue inflammation. J Autoimmun (2013) 40:28–44. 10.1016/j.jaut.2012.07.009 22921334 PMC3524403

[25] Kemper C Chan AC Green JM Brett KA Murphy KM Atkinson JP. Activation of human CD4+ cells with CD3 and CD46 induces a T-regulatory cell 1 phenotype. Nature (2003) 421:388–92. 10.1038/nature01315 12540904

[26] Sutavani RV Bradley RG Ramage JM Jackson AM Durrant LG Spendlove I. CD55 costimulation induces differentiation of a discrete T regulatory type 1 cell population with a stable phenotype. J Immunol (2013) 191:5895–903. 10.4049/jimmunol.1301458 24198281

[27] Barrat FJ Cua DJ Boonstra A Richards DF Crain C Savelkoul HF In vitro generation of interleukin 10-producing regulatory CD4(+) T cells is induced by immunosuppressive drugs and inhibited by T helper type 1 (Th1)- and Th2-inducing cytokines. J Exp Med (2002) 195:603–16. 10.1084/jem.20011629 11877483 PMC2193760

[28] Saidha S Eckstein C Calabresi PA. New and emerging disease modifying therapies for multiple sclerosis. Ann N Y Acad Sci (2012) 1247:117–37. 10.1111/j.1749-6632.2011.06272.x 22224673

[29] Bacchetta R Sartirana C Levings MK Bordignon C Narula S Roncarolo MG. Growth and expansion of human T regulatory type 1 cells are independent from TCR activation but require exogenous cytokines. Eur J Immunol (2002) 32:2237–45. 10.1002/1521-4141(200208)32:8<2237::AID-IMMU2237>3.0.CO;2-2 12209636

[30] Cobbold SP Nolan KF Graca L Castejon R Le Moine A Frewin M Regulatory T cells and dendritic cells in transplantation tolerance: molecular markers and mechanisms. Immunol Rev (2003) 196:109–24. 10.1046/j.1600-065X.2003.00078.x 14617201

[31] Rahmoun M Foussat A Groux H Pene J Yssel H Chanez P. Enhanced frequency of CD18- and CD49b-expressing T cells in peripheral blood of asthmatic patients correlates with disease severity. Int Arch Allergy Immunol (2006) 140:139–49. 10.1159/000092533 16601351

[32] Schuler PJ Saze Z Hong CS Muller L Gillespie DG Cheng D Human CD4+ CD39+ regulatory T cells produce adenosine upon co-expression of surface CD73 or contact with CD73+ exosomes or CD73+ cells. Clin Exp Immunol (2014) 177:531–43. 10.1111/cei.12354 24749746 PMC4226604

[33] Chen L Flies DB. Molecular mechanisms of T cell co-stimulation and co-inhibition. Nat Rev Immunol (2013) 13:227–42. 10.1038/nri3405 23470321 PMC3786574

[34] Thaventhiran T T cell co-inhibitory receptors-functions and signalling mechanisms. J Clin Cell Immunol (2013) 1 10.4172/2155-9899.S12-005

[35] Wang J Okazaki IM Yoshida T Chikuma S Kato Y Nakaki F PD-1 deficiency results in the development of fatal myocarditis in MRL mice. Int Immunol (2010) 22:443–52. 10.1093/intimm/dxq026 20410257

[36] Okazaki T Okazaki IM Wang J Sugiura D Nakaki F Yoshida T PD-1 and LAG-3 inhibitory co-receptors act synergistically to prevent autoimmunity in mice. J Exp Med (2011) 208:395–407. 10.1084/jem.20100466 21300912 PMC3039848

[37] Joller N Hafler JP Brynedal B Kassam N Spoerl S Levin SD Cutting edge: TIGIT has T cell-intrinsic inhibitory functions. J Immunol (2011) 186:1338–42. 10.4049/jimmunol.1003081 21199897 PMC3128994

[38] Fourcade J Sun Z Benallaoua M Guillaume P Luescher IF Sander C Upregulation of Tim-3 and PD-1 expression is associated with tumor antigen-specific CD8+ T cell dysfunction in melanoma patients. J Exp Med (2010) 207:2175–86. 10.1084/jem.20100637 20819923 PMC2947081

[39] Anderson AC. Tim-3: an emerging target in the cancer immunotherapy landscape. Cancer Immunol Res (2014) 2:393–8. 10.1158/2326-6066.CIR-14-0039 24795351

[40] Gros A Robbins PF Yao X Li YF Turcotte S Tran E PD-1 identifies the patient-specific CD8(+) tumor-reactive repertoire infiltrating human tumors. J Clin Invest (2014) 124:2246–59. 10.1172/JCI73639 24667641 PMC4001555

[41] Francisco LM Sage PT Sharpe AH. The PD-1 pathway in tolerance and autoimmunity. Immunol Rev (2010) 236:219–42. 10.1111/j.1600-065X.2010.00923.x 20636820 PMC2919275

[42] Wang J Yoshida T Nakaki F Hiai H Okazaki T Honjo T. Establishment of NOD-Pdcd1-/- mice as an efficient animal model of type I diabetes. Proc Natl Acad Sci U S A (2005) 102:11823–8. 10.1073/pnas.0505497102 16087865 PMC1188011

[43] Hanazawa A Hayashizaki K Shinoda K Yagita H Okumura K Lohning M CD49b-dependent establishment of T helper cell memory. Immunol Cell Biol (2013) 91:524–31. 10.1038/icb.2013.36 23897120

[44] Sakuishi K Jayaraman P Behar SM Anderson AC Kuchroo VK. Emerging Tim-3 functions in antimicrobial and tumor immunity. Trends Immunol (2011) 32:345–9. 10.1016/j.it.2011.05.003 21697013 PMC3164311

[45] Joller N Lozano E Burkett PR Patel B Xiao S Zhu C Treg cells expressing the coinhibitory molecule TIGIT selectively inhibit proinflammatory Th1 and Th17 cell responses. Immunity (2014) 40:569–81. 10.1016/j.immuni.2014.02.012 24745333 PMC4070748

[46] Gautron AS Dominguez-Villar M De Marcken M Hafler DA. Enhanced suppressor function of TIM-3+ FoxP3+ regulatory T cells. Eur J Immunol (2014) 44:2703–11. 10.1002/eji.201344392 24838857 PMC4165702

[47] Vicente R Quentin J Mausset-Bonnefont AL Chuchana P Martire D Cren M Nonclassical CD4+CD49b+ regulatory T cells as a better alternative to conventional CD4+CD25+ T cells to dampen arthritis severity. J Immunol (2016) 196:298–309. 10.4049/jimmunol.1501069 26590312

[48] Mazza G Sabatos-Peyton CA Protheroe RE Herman A Campbell JD Wraith DC. Isolation and characterization of human interleukin-10-secreting T cells from peripheral blood. Hum Immunol (2010) 71:225–34. 10.1016/j.humimm.2009.12.003 20034527 PMC3399767

[49] Bae J Lee SJ Park CG Lee YS Chun T. Trafficking of LAG-3 to the surface on activated T cells via its cytoplasmic domain and protein kinase C signaling. J Immunol (2014) 193:3101–12. 10.4049/jimmunol.1401025 25108024

[50] Huang YH Zhu C Kondo Y Anderson AC Gandhi A Russell A CEACAM1 regulates TIM-3-mediated tolerance and exhaustion. Nature (2015) 517:386–90. 10.1038/nature13848 25363763 PMC4297519

[51] Goncalves Silva I Gibbs BF Bardelli M Varani L Sumbayev VV. Differential expression and biochemical activity of the immune receptor Tim-3 in healthy and malignant human myeloid cells. Oncotarget (2015) 6:33823–33. 10.18632/oncotarget.5257 26413815 PMC4741805

[52] Nagaishi T Chen Z Chen L Iijima H Nakajima A Blumberg RS. CEACAM1 and the regulation of mucosal inflammation. Mucosal Immunol (2008) 1(Suppl 1):S39–42. 10.1038/mi.2008.50 19079227 PMC3901578

[53] Grossman WJ Verbsky JW Tollefsen BL Kemper C Atkinson JP Ley TJ. Differential expression of granzymes A and B in human cytotoxic lymphocyte subsets and T regulatory cells. Blood (2004) 104:2840–8. 10.1182/blood-2004-03-0859 15238416

[54] Anderson PO Manzo BA Sundstedt A Minaee S Symonds A Khalid S Persistent antigenic stimulation alters the transcription program in T cells, resulting in antigen-specific tolerance. Eur J Immunol (2006) 36:1374–85. 10.1002/eji.200635883 16708405 PMC2652694

[55] Gandhi R Kumar D Burns EJ Nadeau M Dake B Laroni A Activation of the aryl hydrocarbon receptor induces human type 1 regulatory T cell-like and Foxp3(+) regulatory T cells. Nat Immunol (2010) 11:846–53. 10.1038/ni.1915 20676092 PMC2929008

[56] Schmetterer KG Neunkirchner A Wojta-Stremayr D Leitner J Steinberger P Pickl WF. STAT3 governs hyporesponsiveness and granzyme B-dependent suppressive capacity in human CD4+ T cells. FASEB J (2015) 29:759–71. 10.1096/fj.14-257584 25398767 PMC4422363

[57] Takeuchi A Badr Mel S Miyauchi K Ishihara C Onishi R Guo Z CRTAM determines the CD4+ cytotoxic T lymphocyte lineage. J Exp Med (2016) 213:123–38. 10.1084/jem.20150519 26694968 PMC4710199

[58] Hill EV Ng TH Burton BR Oakley CM Malik K Wraith DC. Glycogen synthase kinase-3 controls IL-10 expression in CD4(+) effector T-cell subsets through epigenetic modification of the IL-10 promoter. Eur J Immunol (2015) 45:1103–15. 10.1002/eji.201444661 25627813 PMC4405077

[59] Dong J Ivascu C Chang HD Wu P Angeli R Maggi L IL-10 is excluded from the functional cytokine memory of human CD4+ memory T lymphocytes. J Immunol (2007) 179:2389–96. 10.4049/jimmunol.179.4.2389 17675500

